# The Rat Thoracic Ultrasound protocol: scanning technique and normal findings

**DOI:** 10.3389/fvets.2024.1286614

**Published:** 2024-02-19

**Authors:** Anna Piskovská, Katarzyna Kraszewska, Karel Hauptman, Vladimír Jekl

**Affiliations:** ^1^Jekl and Hauptman Veterinary Clinic, Brno, Czechia; ^2^Department of Pharmacology and Pharmacy, Faculty of Veterinary Medicine, VETUNI, Brno, Czechia; ^3^Vetcardia Veterinary Clinic, Warsaw, Poland

**Keywords:** pleura, ultrasonography, rat, RATTUS, lung, respiratory disease, thorax

## Abstract

Respiratory diseases (especially pneumonia) are very common disorders in pet rats. The suspected diagnosis is mostly based on the clinical signs, thoracic auscultation, and thoracic radiography. However, auscultation is insensitive in determining the severity of the disease, and radiographs are often unremarkable. Non-cardiac thoracic ultrasonography is increasingly used in veterinary medicine; however, it has not been described in detail in rats. Thoracic ultrasonic examination was conducted on 400 client-owned conscious pet rats. The rats were examined in the period from June 2023 to August 2023 in two veterinary clinics. Due to the small size of the animal, different anatomical considerations, and different evaluation protocols, as well as to meet the optimal outcome of detailed thoracic ultrasound, a standard methodological protocol was developed, and the name RATTUS (Rat Thoracic Ultrasound) was proposed. Typical signs of normal RATTUS were described (bat sign, lung sliding, A-lines, abdominal curtain sign, ski jump sign, lung pulse, seashore sign in M-mode, and bamboo sign). The new evaluation of lung inflation symmetry by substernal access was also described. The methodical approach presented and the normal findings description are proposed to be used for a standard/routine thoracic ultrasound examination in pet rats.

## Introduction

Non-cardiac thoracic ultrasonography is one of the increasingly used imaging methods in veterinary medicine. It complements radiography in the examination of patients with diseases of the chest wall, pleural space, mediastinum, and lung. Ultrasound wave penetration is hampered by gas, such as is found in the aerated lung. Therefore, pleural and lung ultrasound is about evaluating the absence or presence of gas by specific artefacts (1). Different aerations of damaged lungs generate various artefacts that can be used for diagnostic evaluation. Thoracic ultrasound can be a useful diagnostic step in cases where there is fluid in the thoracic cavity, creating soft tissue opacities on the lung radiograph indistinguishable from other conditions (e.g., a thoracic mass). Pleural pathological conditions (pleural effusion and pleural thickening), lung consolidations, and artefact detection can be easily visualised by an ultrasonographic examination for the diagnosis of various thoracic diseases (2, 3).

Thoracic ultrasound is preferably performed on conscious animals as anaesthesia may cause artefacts that could be misinterpreted as pathological findings (e.g., gravitational atelectasis) (3–5). An ‘evaluation key’ standardly used in the thoracic ultrasound of dogs and cats includes: bat sign, A-lines, lung sliding, curtain sign, lung pulse, ski jump sign, and B-lines. More information is given in Supplementary material (3–19).

Ma et al. (20) described lung ultrasonography in an experimental rat model. However, the animals were examined under pentobarbital sodium anaesthesia in a supine position, and only limited ultrasonographic findings were reported.

The aim of the present study is to describe the methodical approach and normal findings of thoracic ultrasound in healthy pet rats.

## Materials and methods

### Animals

In total, 400 client-owned pet rats were examined in the period from June 2022 to August 2023. The body weight ranged from 260 g to 885 g (mean ± SD 467.1 ± 119.8 g), and the age ranged from 3 months to 30 months (20 ± 4.6 months).

### Equipment

A multifrequency linear probe (8–14 MHz, SonoScape, S22, China, with MI 0.5–0.7, TIS 0.1) was used for the thoracic cavity evaluation. Due to the size of the rats, a micro-convex probe (9–14 MHz, SonoScape, S22, China, with MI 0.7, TIS 0.2) was used only to evaluate the presence of B-lines. For the identification of lung surface artefacts, pleural line, and lung sliding, a special ‘lung preset’ was utilised. This preset had harmonics turned off, used the lowest frequency of the probe (8 MHz) with persistence turned to zero, a focal position set at the level of the pleural line, and used an increased time gain compensation (TGC) at the distal (far) field of the screen. Such settings create a ‘coarser’ picture. The examination was conducted in two-dimensional (2D) mode and in M-mode. In all animals, video loops and images were saved routinely for possible further evaluation.

### Animal preparation

The examination was conducted on conscious (non-sedated) client-owned pet rats. In most animals, it was not necessary to shave the hair if a generous amount of ultrasound gel was used. The examiner carefully restrained the animal with one hand whilst the other hand held the probe.

### Rat Thoracic Ultrasound methodology

Thoracic cavity evaluation was conducted by two operators (KK—an experienced clinician, with over 10 years of experience in performing echocardiographic examinations and 5 years of experience in lung ultrasound with advanced training from human lung ultrasound experts, and AP, with over 1 year of lung ultrasound examination training by KK and human lung ultrasound experts).

The examination protocol followed thoracic ultrasound, as described in dogs and cats (3). For better understanding, a detailed description of ultrasonographic findings (‘evaluation key’) is presented in Supplementary material.

**Figure 1:**
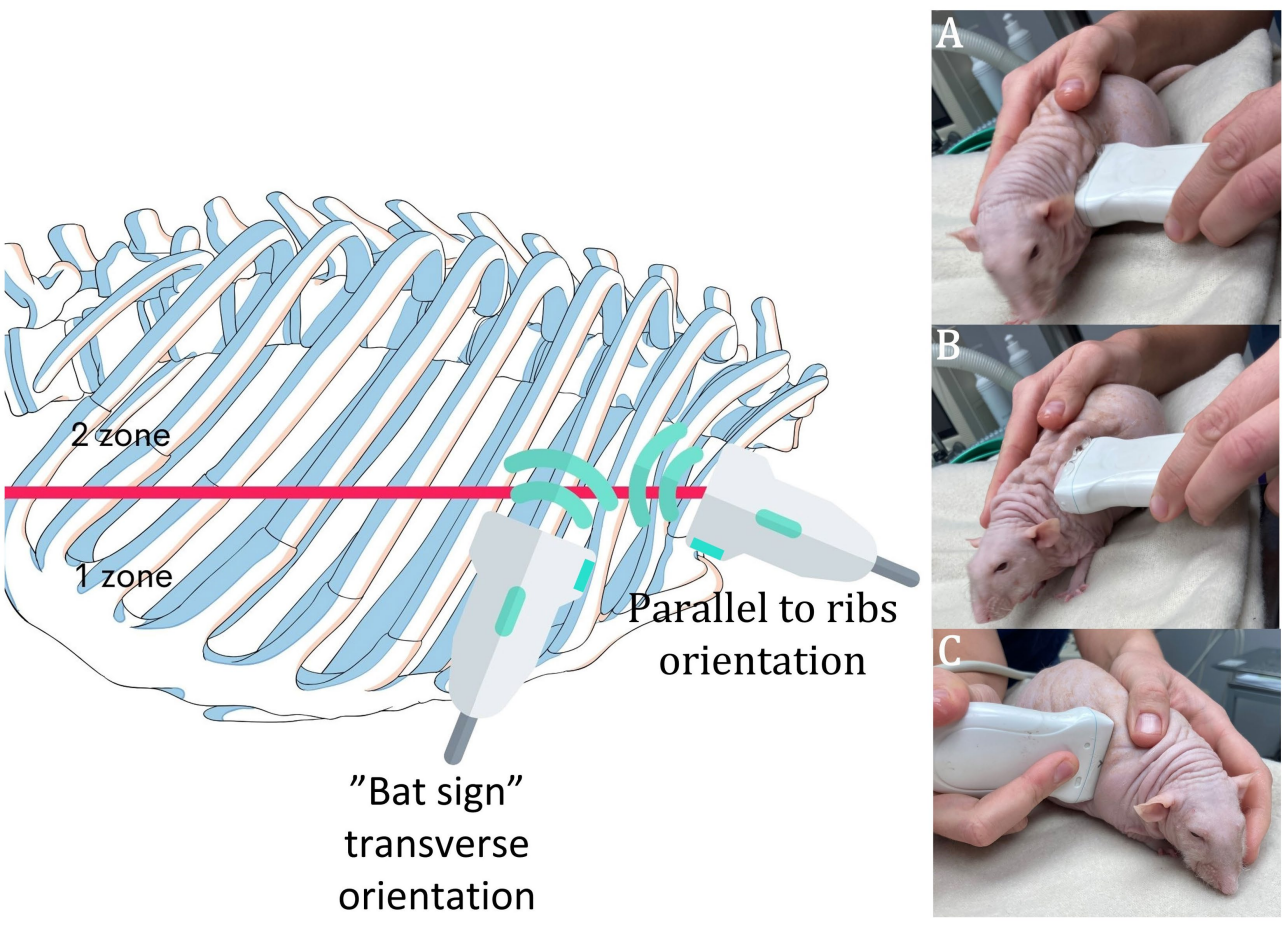
RATTUS performance. The rat was placed on a towel with careful restraint, and the fur was not shaved. We used a lot of gel to obtain ideal contact. The examination commenced with the probe being placed perpendicular to the ribs and the marker pointing cranially (**A**—examination of the axillary line, first zone, **B**—examination of the scapular line, second zone—as is visible in the picture, both zones were examined without changing the placement of the probe). Then, the probe was turned 90° with the marker pointing towards the sternum **(C)**. The whole thorax was clearly scanned. For a better demonstration, a Dumbo fuzz (hairless) rat was used.

**Figure 2:**
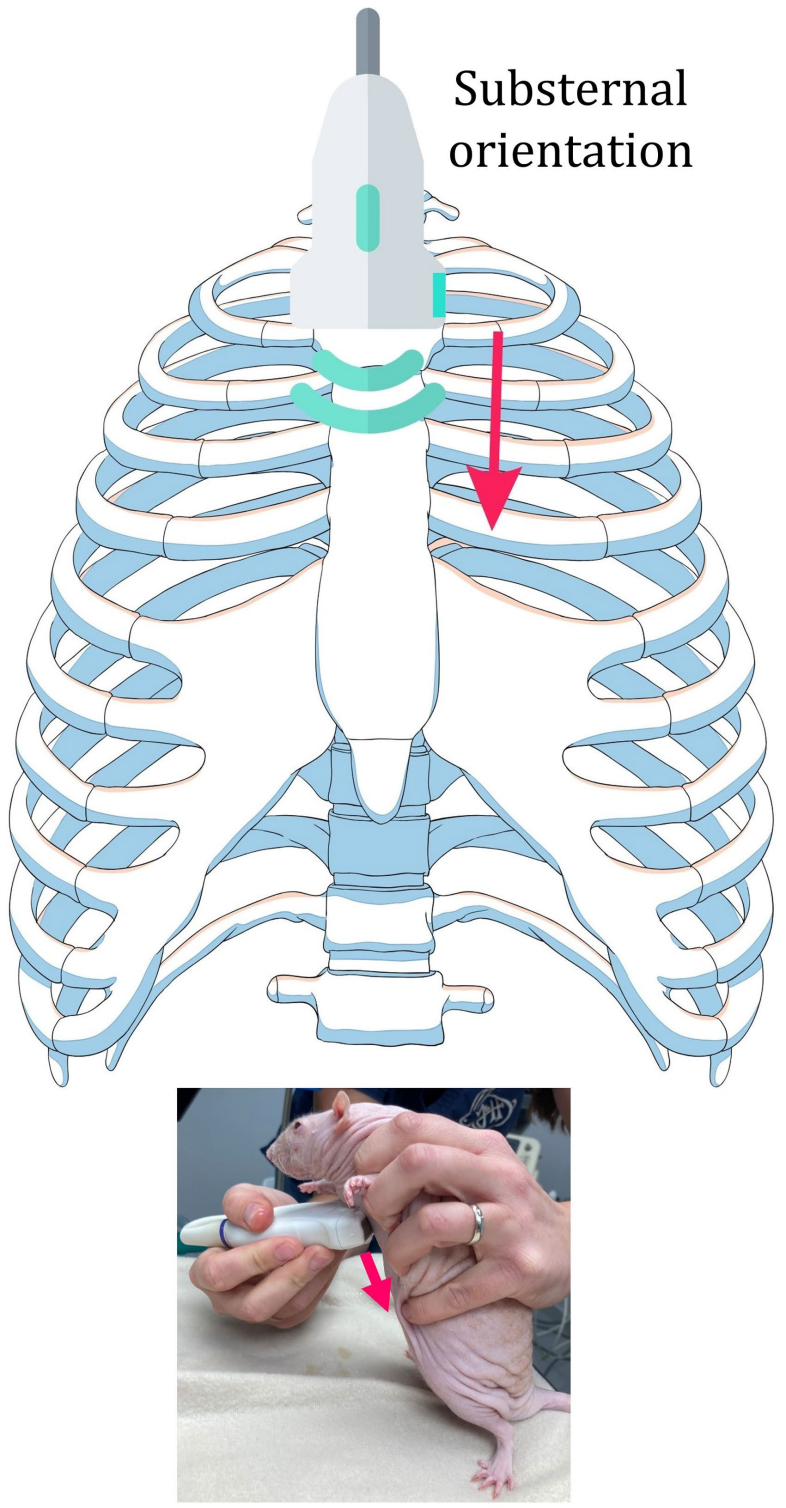
Substernal access. The probe was placed perpendicularly to the sternum, with the sternum in the middle of the probe surface. The probe was moved from the cranial border (brachial muscles) to the caudal border (abdominal curtain sign) with special attention to the area of the heart. The marker was placed on the left side to obtain a standardised view.

Due to the small size of the animals, different anatomical considerations, and different evaluation protocols, as well as to meet the optimal outcome of detailed thoracic ultrasound, a standard methodological protocol was developed, and the methodology name RATTUS (Rat Thoracic Ultrasound) was proposed. The RATTUS evaluation commenced with the ultrasonic probe being placed perpendicular to the ribs, with the marker pointing to the head. The probe was then rotated 90° with the marker pointing to the sternum (Figure 1). The last access involved placing the probe substernally to evaluate the lung inflation symmetry (Figure 2).

To avoid missing a thoracic pathology, it was essential to examine the entire thoracic cavity. To be sure that the whole thoracic cavity was precisely examined, scanning must be performed bordered by exact anatomical structures. The cranial border of the thoracic cavity was formed by the brachial muscles, and the caudal border was created by visualising the abdominal curtain sign. The curtain sign is created when the lung is filled with air and it expands and retracts over abdominal organs, obscuring the sonographic view into them. When the probe is moved from the dorsal border to the ventral border, the curtain sign needs to be precisely assessed to evaluate the abnormalities. Then, the pericardio-diaphragmatic region was visualised, and sternal and pectoral muscles were evaluated. At the ventral border of the thoracic cavity, a precise examination of the cranial mediastinum was required. The mediastinum was examined to rule out the presence of a neoplastic mass (e.g., lymphoma) (21), and then the subjective assessment of the heart anatomy and function was performed. The dorsal border of the thoracic cavity delineates the hypaxial musculature. The same scheme was used for both sides of the thoracic cavity.

To perform a repeatable examination, each hemithorax was divided into two zones: the first extended from the axillary line to the scapular line, and the second from the scapular line to the paravertebral line (Figure 1). Typically, those two zones could be examined from one body–probe contact place due to the adequate subcutis space, which enabled easy movement of the probe from the cranial border to the caudal border and from the ventral border to the dorsal border without changing the location of the probe. Those zones (axillary zone—first zone and scapular zone—second zone) were used to better describe the location of the found pathologies.

The substernal access was used to assess the symmetry of the lung inflation. The probe was placed perpendicular to the sternum, with the sternum in the centre of the probe surface. The probe was then moved from the cranial to the caudal border, with special attention to the area of the heart. Attention must be given to the precise placement of the probe on the sternum because oblique placement of the probe might create a false-positive heart shift.

## Results

Rats were considered healthy which were not dyspneic and had normal lung and heart auscultation (no crackles or other pathological sounds were detected). In all of the healthy rats, normal ultrasonographic findings were observed.

A complete thoracic ultrasonography was possible in all pet rats. Most rats calmed down after being restless at the beginning of the examination and were easily examined in the sternal recumbency. The duration of the examination was from 10 to 15 min according to the compliance of the animal.

### RATTUS normal findings

The pleural line was a regular, hyperechoic line that moved synchronously with respiration.

**Figure 3:**
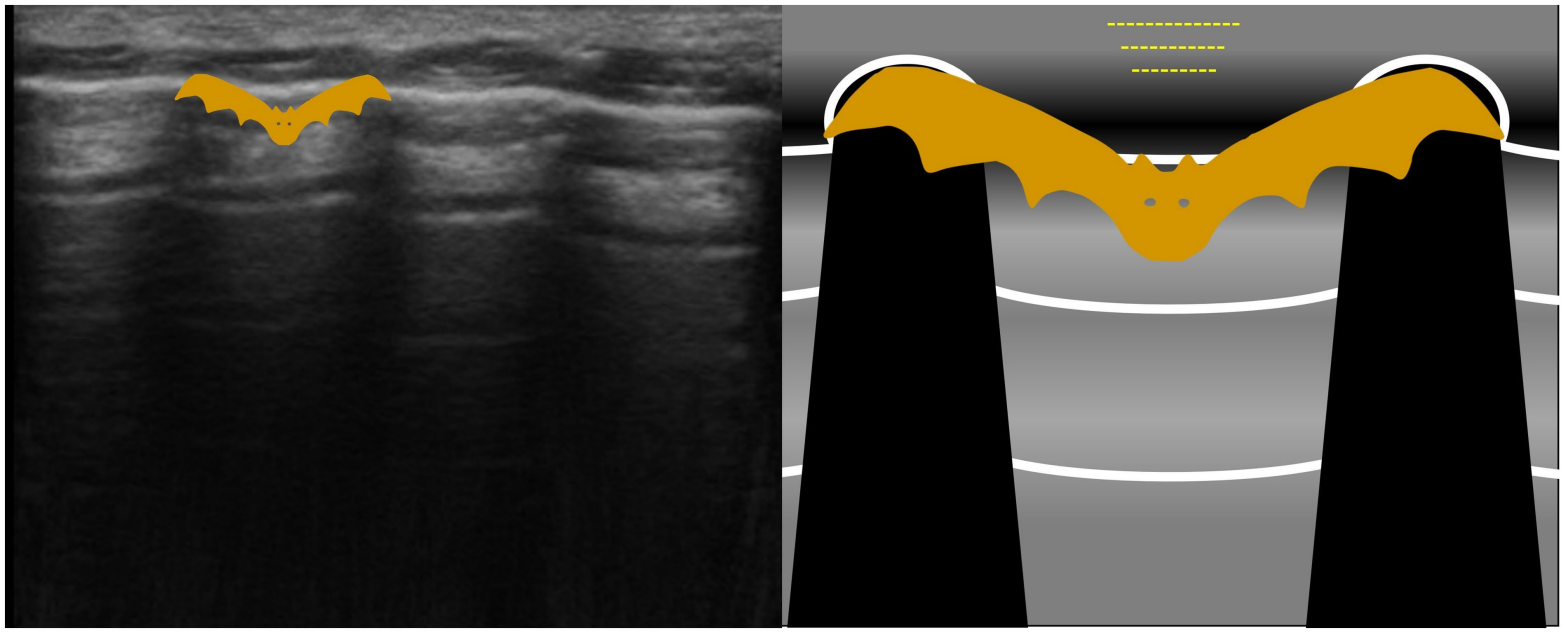
The bat sign is an abstract sign established to help the examiner with lung surface orientation. The image obtained is called a ‘bat sign’, as the rib heads and pleural line resemble the wings and body of a bat.

The bat sign (Figure 3; Supplementary Video 1) consisted of a rib surface and pleural line. Due to the size of a rat, the ribs were seen as round structures even when the probe was aimed transversally, as the probe size prevented a clear view of the intercostal space between two ribs.

**Figure 4:**
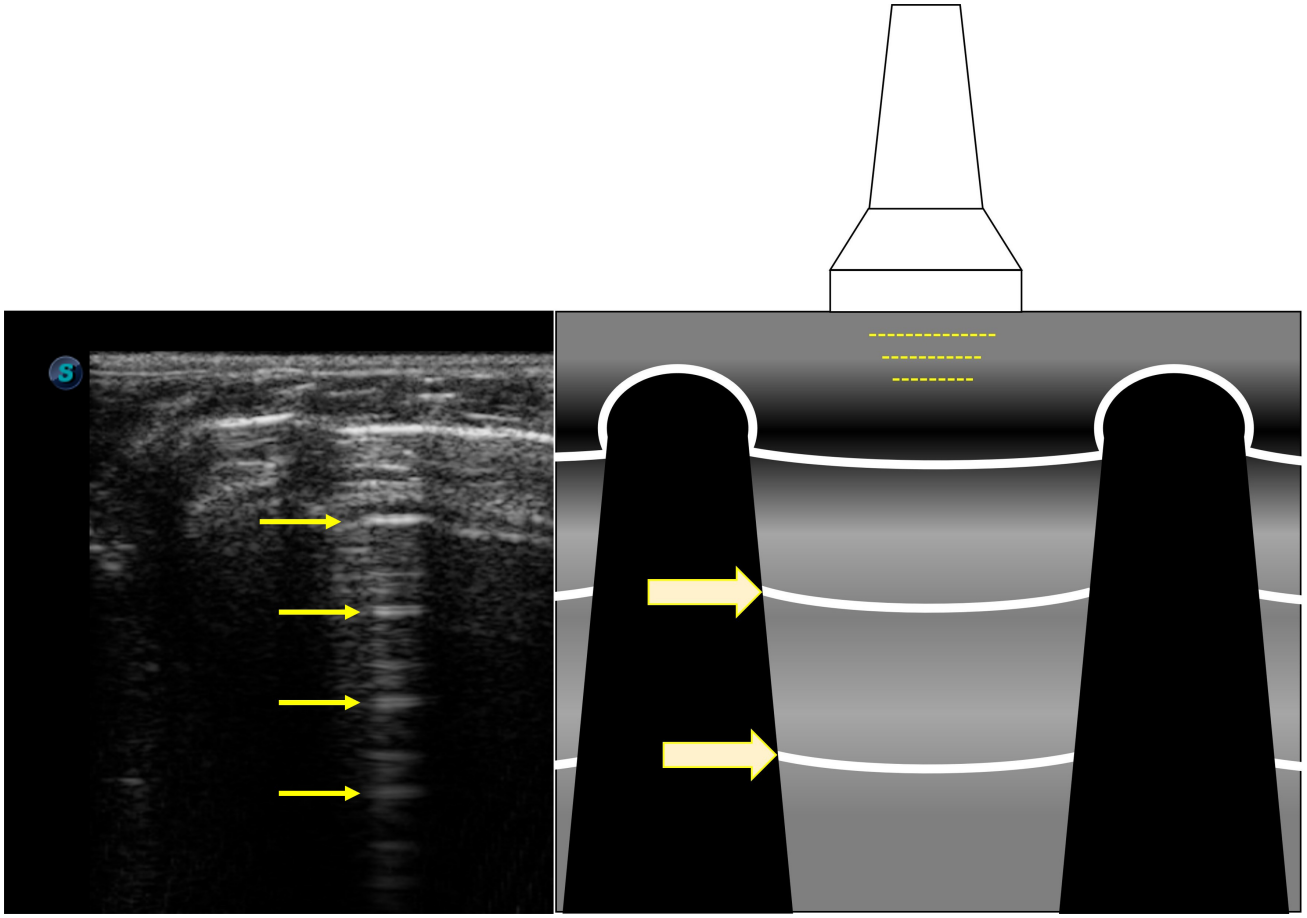
A-lines are hyperechoic horizontal lines (yellow arrows) and are reverberation artefacts of the pleural line.

A-lines were easily visualised as hyperechoic horizontal lines (Figure 4; Supplementary Video 1).

The key to identifying lung sliding with RATTUS was to focus on the pleural line and ignore other parts of the sonogram (Supplementary Video 1). The animal and probe must be as still as possible, and colour Doppler imaging might be used to help detect the presence of lung sliding, but this technique is challenging in active rats when the movements of an animal create colour Doppler artefacts.

**Figure 5:**
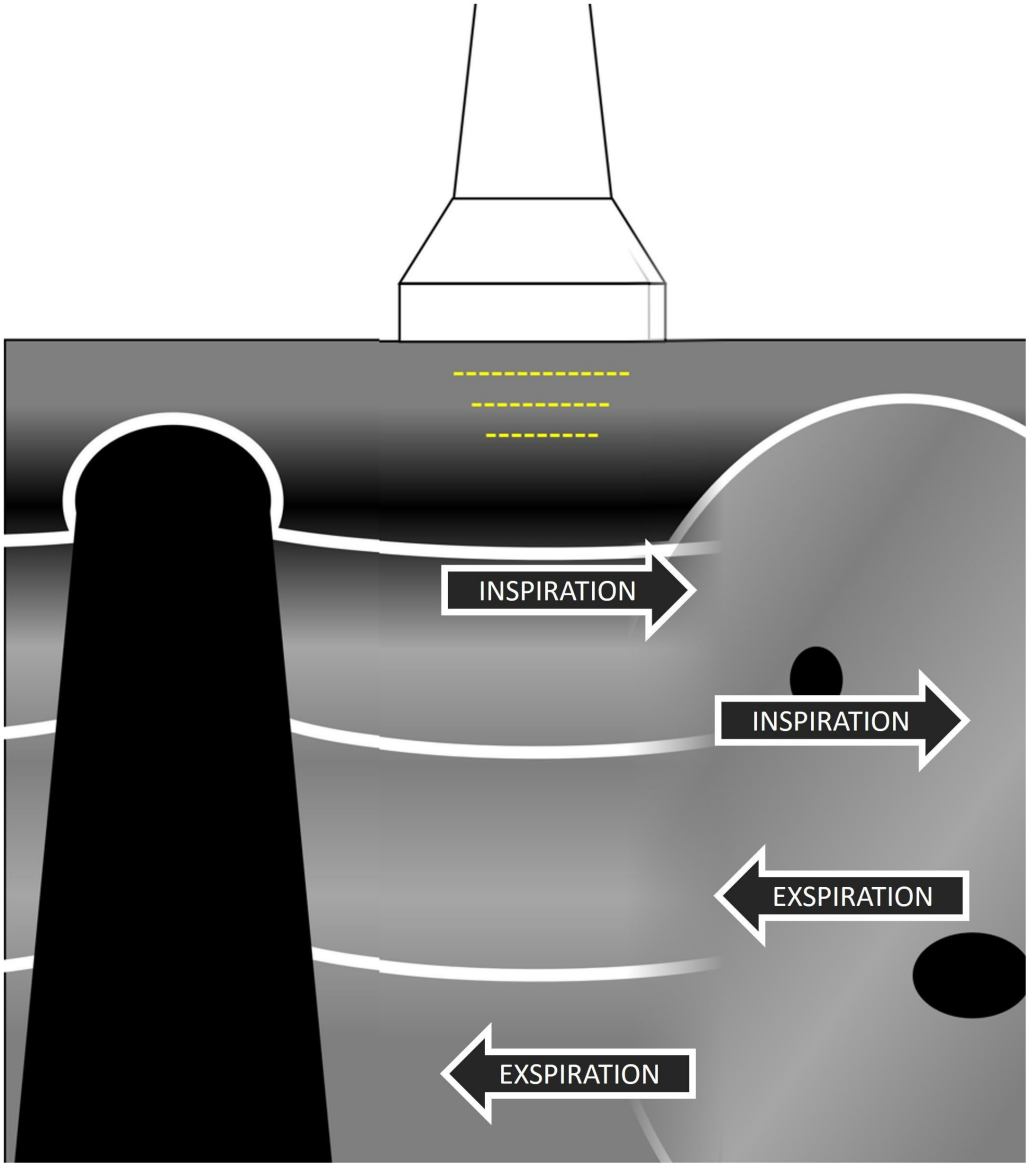
The abdominal curtain sign is visualised when the lung contracts over mediastinal or abdominal organs and hides the sonographic view into them as a curtain hides a window; hidden organs are again visible when the lung contracts during expiration.

In rats, the heart curtain sign was barely visible, and the abdominal curtain sign was only a slight suggestion of the curtain sign, which is usually seen in larger animals (Figure 5; Supplementary Video 2). It was always necessary to concentrate on the normal curtain sign, and in hyperpnoeic animals, slow motion evaluation might be necessary because, contrary to larger animals, the lungs overlap the abdominal content only in a very small region.

**Figure 6:**
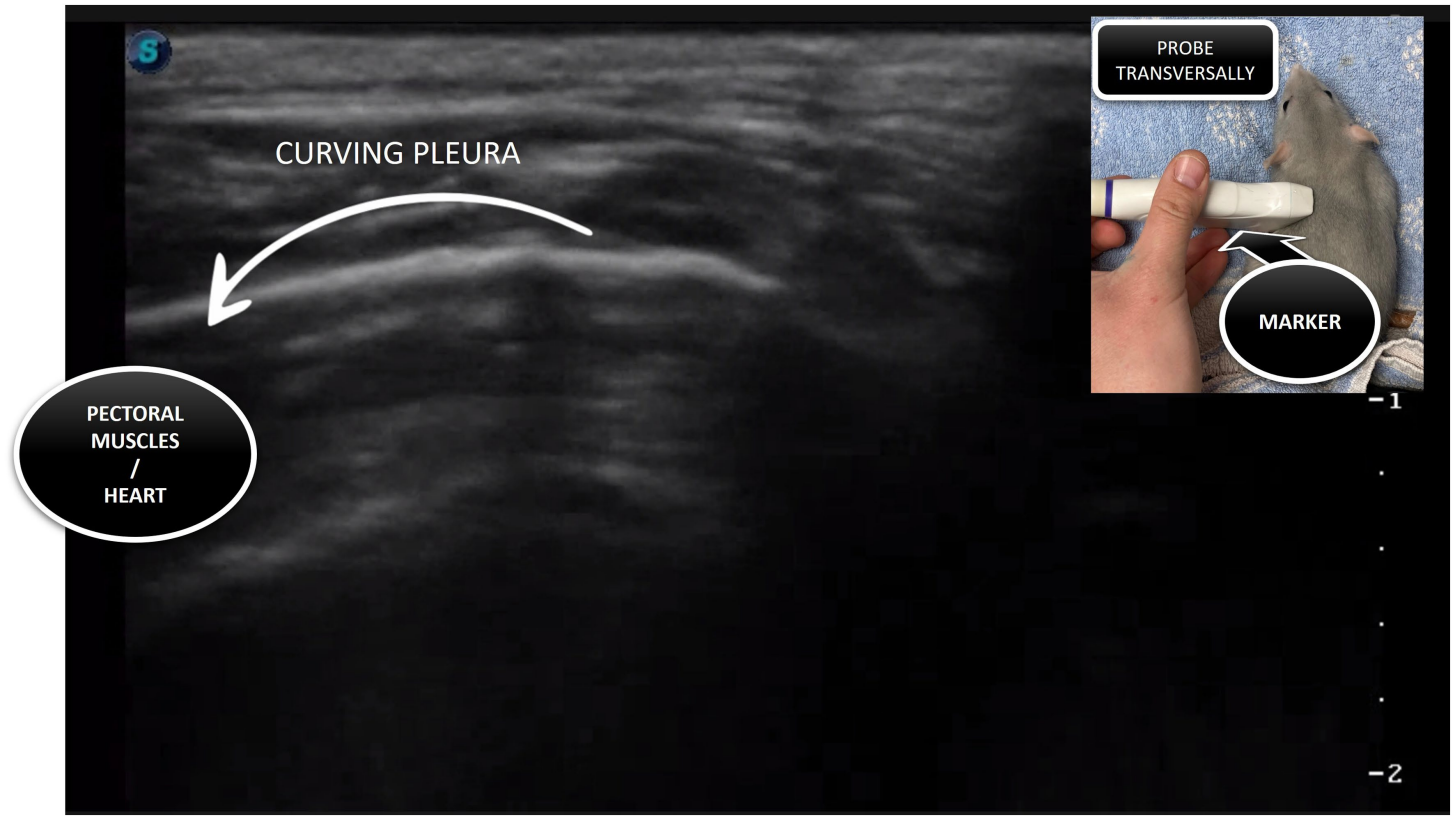
The ski jump sign is visualised when the probe is placed parallel to the ribs, with the lung curving away from the sonogram, and this creates a sonographic picture like a ski jump.

The ski jump sign (Figure 6; Supplementary Video 3) was used to rule out small volumes of pleural effusion that may be missed in small rats with the probe-oriented transverse to the ribs. When the ski jump sign was evaluated in rats, the probe was oriented parallel to the ribs, and it was necessary to concentrate on the curving part of the pleura as the small amount of pleural effusion can create a slight suggestion of ‘sailing’ pleura, confirming the presence of the small amount of fluid.

The lung pulse in a rat is usually best displayed in the lobes close to the heart (Supplementary Video 4), confirming that the pleura is in contact.

**Figure 7:**
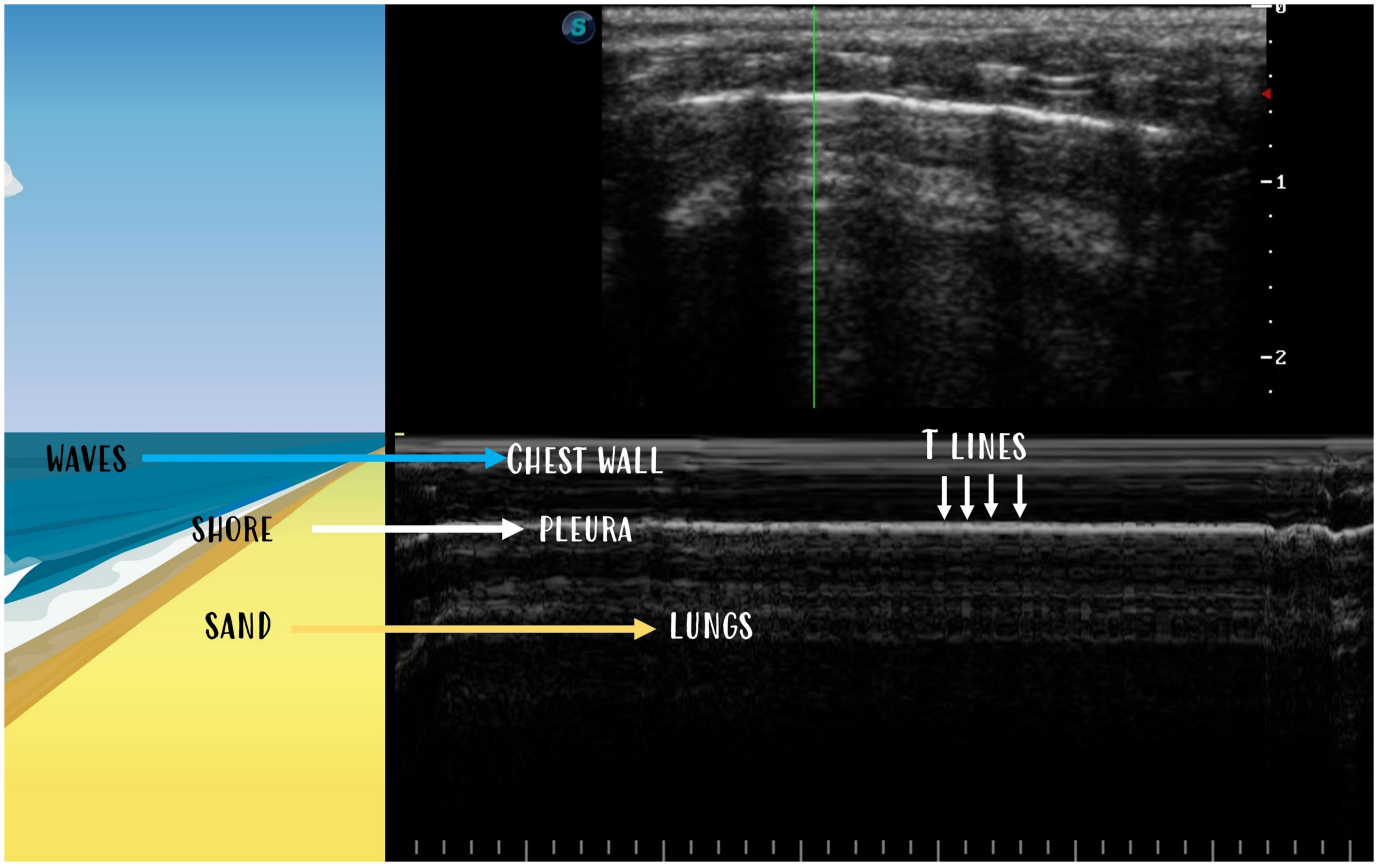
The seashore sign is visualised in M-mode when pleura creates a horizontal hyperechoic line (the border between waves and sand). The aerated lung creates a sandy appearance, and the chest wall lying above creates stratifications. T-lines are vertical lines going from the pleural line to the bottom.

The seashore sign (Figure 7) is a visualisation of the lung sliding using M-mode, and it is conducted on rats. To perform an M-mode examination in a rat, the sweep speed on the ultrasound machine needs to be reduced to a minimum. The pleura produces a horizontal hyperechoic line (representing the border between waves and sand). An aerated lung creates a sandy appearance, and the overlying chest wall creates stratifications. Considering that RATTUS is performed without anaesthesia, M-mode evaluation was often very challenging due to the animal’s movement and respiratory rate.

**Figure 8:**
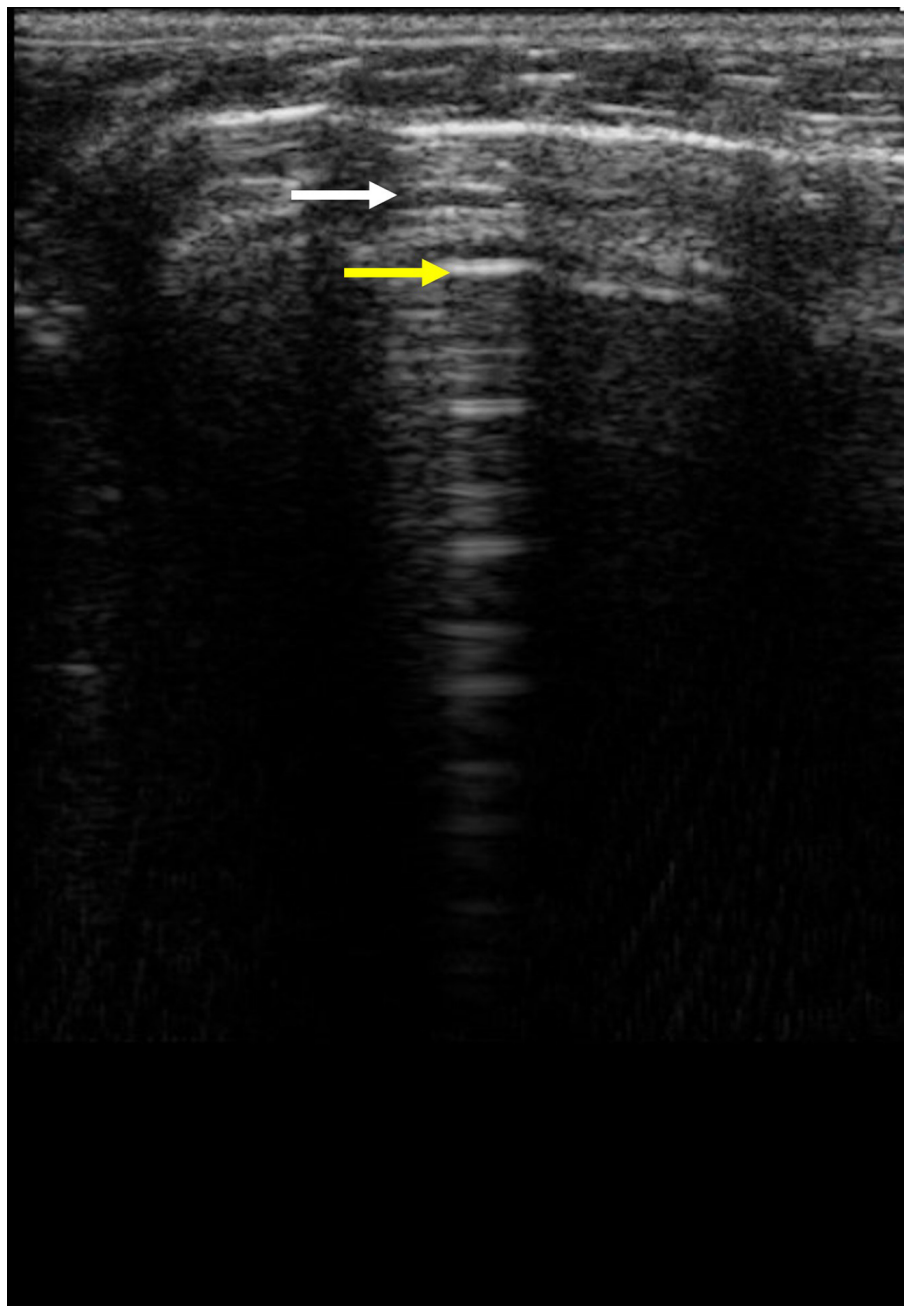
The bamboo sign is the sign when, between two A-lines, a smaller hypoechoic horizontal artefact is visible and is called sub-A lines or Pi-lines (white arrow, A-lines yellow arrow).

**Figure 9:**
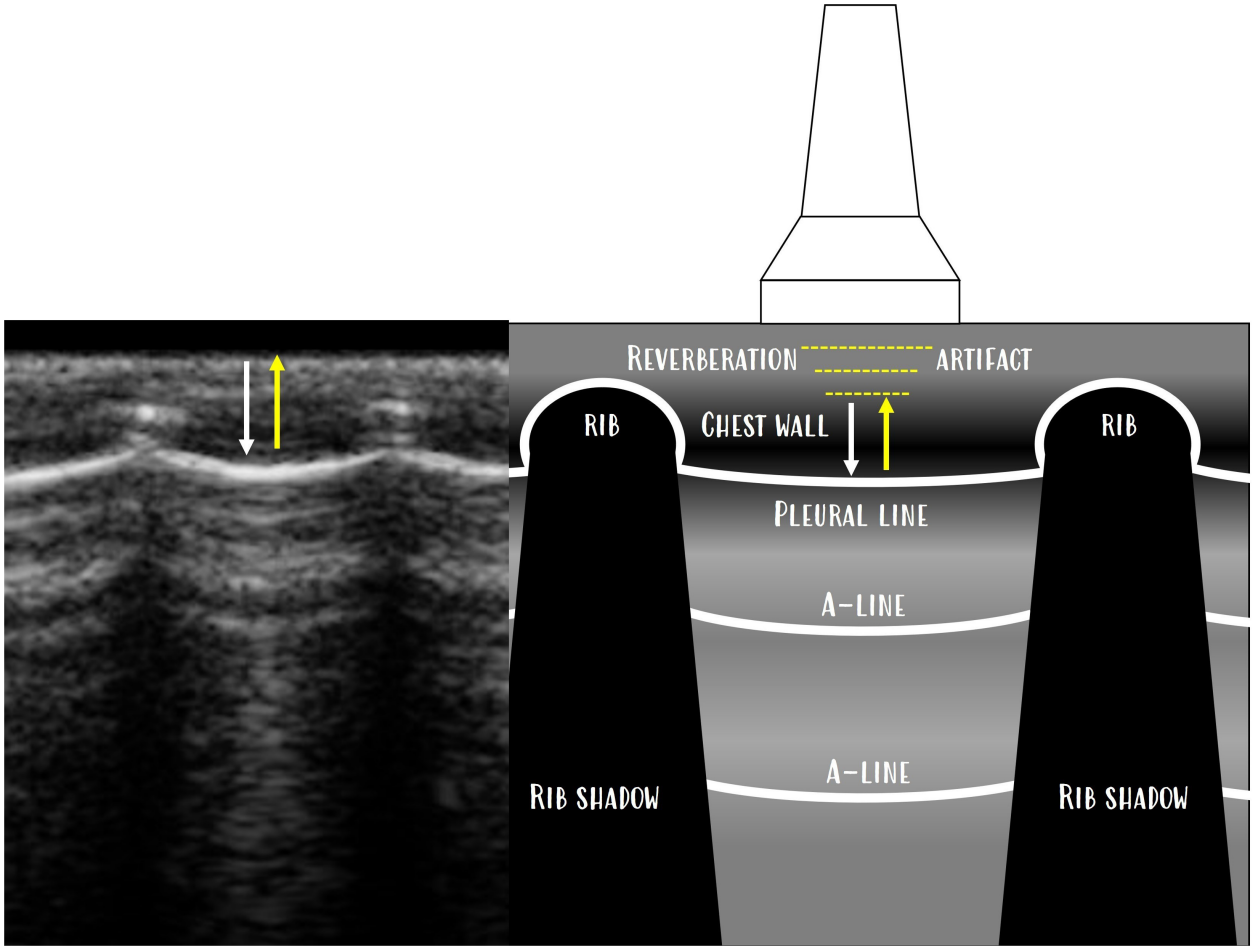
Reverberation artefact, where the subpleural line does not show anatomical lung tissue but a reverberation artefact of the chest wall is displayed above the pleura as A-lines. A-lines are reverberation artefacts of the pleura, and the space between the pleura and A-lines and the space between each A-line is a reverberation artefact of the chest wall.

In rats, the bamboo sign (Figure 8) artefact is seen even in animals with normal body conditions. This is probably due to the lower amount of fat in the subcutaneous tissue, which creates more reverberation (Figure 9).

The substernal access is used for the evaluation of the lung inflation symmetry (Supplementary Video 5). When the sternum was in the middle of the sonogram, both lungs were in the same line, moving synchronously with respiration. The position of the marker pointed to the left side to create a standard and repeatable protocol. Than the left side of the thoracic cavity agreed with the left side of the saved loop for better and standardized orientation.

**Figure 10:**
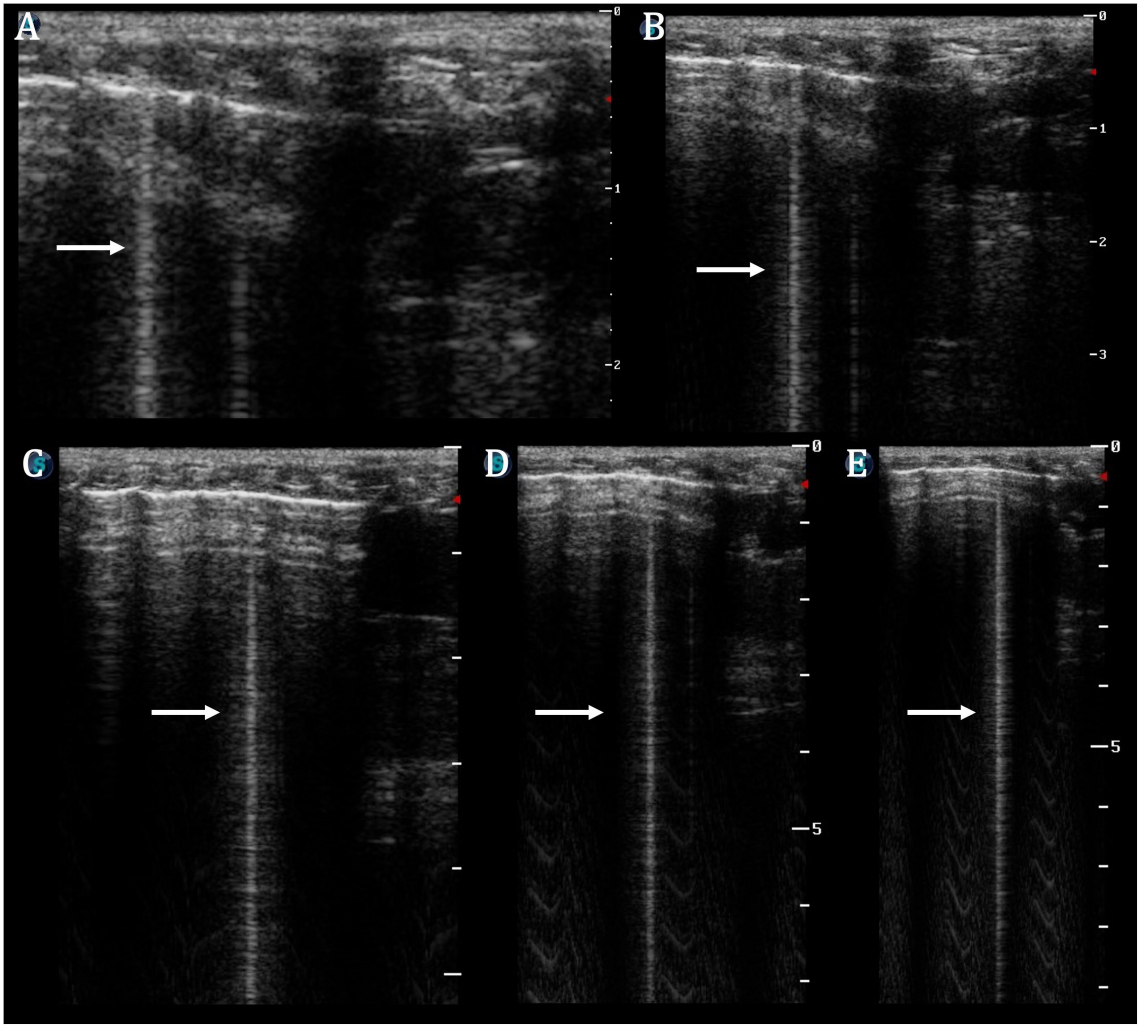
Single B-line (arrow) on linear probe proven by changing depth (**A−E**—**A**: depth 2 cm, **E**: depth 9 cm). The micro-convex probe is usually recommended for vertical artefact assessment; however, by increasing the depth of the image, there is the ability to confirm that B-lines are ‘true’ B-lines and did not terminate before reaching the far field of the ultrasound image.

Occasionally, single B-lines were identified. As the examination was conducted with a linear probe in all cases of B-lines, the changing of the depth was used to confirm that B-lines are ‘true’ B-lines (Figure 10). Less than two B-lines were considered a normal/physiological finding (20).

## Discussion

Respiratory diseases (especially pneumonia) are very common disorders in pet rats (22, 23). The suspected diagnosis is mostly based on the clinical signs, thoracic auscultation, and thoracic radiography. However, the auscultation is insensitive to determining the severity of the disease, and radiographs are commonly unremarkable (24). A computed tomography examination seems to be promising in the diagnosis of intrathoracic disease (25) however, this examination is still not available in many veterinary practices.

In recent years, pleura and lung ultrasound have become important tools in human and veterinary medicine (3, 25). It has been shown to be a suitable imaging method for the diagnosis of pulmonary oedema (26–28), pneumonia (29), and pneumothorax (30, 31). Many studies have shown thoracic sonography to be the preferred method for identifying lung and pleural pathological findings when compared to computed tomography, especially due to the lack of radiation exposure and similar examination results. Furthermore, the advantage of thoracic ultrasound is the possibility of performing it ‘bed side’, in contrast to computed tomography (CT) or thoracic X-rays (TXR) (32–36). In RATTUS, the major advantage is the possibility of performing this examination in conscious animals and in normal sternal recumbency.

The thoracic ultrasound was mainly described in rodent experimental studies (2, 20, 37, 38). In mice, lung ultrasound has been successfully used to assess pulmonary congestion in a preclinical model of heart failure (39). In companion rats, only a few studies and clinical case reports of thoracic diseases have been published; however, none of them used thoracic ultrasonography as a diagnostic method (21, 40–44). On the contrary, thoracic ultrasound in companion animals is a standard and well-described diagnostic tool for the diagnosis of cardiogenic oedema (45, 46), pneumothorax (3, 47), pleural effusion (3, 48, 49), or pneumonia (50, 51).

One of the limitations of this examination is the presence of pathological conditions outside the lung periphery, which may be missed due to the aerated lung causing a ‘physiological’ reverberation artefact (20, 52). Another limitation may be rib shadowing, where smaller pathologies may remain hidden (this issue is partially decreased using the transversal view of intercostal spaces and the fact that most clinically manifested disorders occur in larger areas) (52). Due to these limitations, other imaging techniques such as CT and TXR could be useful to provide a complete thoracic cavity overview.

Pleura and lung ultrasound are considered to be a safe, cost-effective, and easily implemented imaging modality that can aid in the assessment of many thoracic pathological findings. It has been shown to improve procedural efficiency whilst decreasing complications, increasing success, and reducing financial strain (53). In the medical treatment of pet rats, the main advantage of RATTUS is the possibility of evaluating the thoracic wall without anaesthesia (in contrast to TXR or CT) (43). Due to the size of the thoracic cavity and the use of a linear probe, the examination is relatively quick and easily conducted. Another advantage of this technique is the repeatability of the examination, which enables the evaluation of the progression of the disease and/or the efficacy of therapy. However, we have not assessed the repeatability of the RATTUS protocol.

The RATTUS protocol and normal ultrasonographic findings reported in the present study may be useful for a routine thoracic examination in pet rats. Further studies in acute and chronic cases of respiratory distress are needed.

## Data availability statement

The raw data supporting the conclusions of this article will be made available by the authors, without undue reservation.

## Ethics statement

Ethical approval was not required for the studies involving animals in accordance with the local legislation and institutional requirements because RATTUS is a non-invasive, non-painful method performed on unanaesthetised pet rats. Written informed consent was obtained from the owners for the participation of their animals in this study.

## Author contributions

AP: Methodology, Writing – original draft. KK: Methodology, Supervision, Writing – review & editing. KH: Supervision, Writing – review & editing. VJ: Conceptualization, Supervision, Validation, Writing – review & editing.

## Supplementary material

SUPPLEMENTARY VIDEO 1: Physiological sonogram https://1drv.ms/v/s!AglSmr1cGU9agRcvHFeUBQ7BLkVWhttps://1drv.ms/v/s!AglSmr1cGU9agRcvHFeUBQ7BLkVWhttps://1drv.ms/v/s!AglSmr1cGU9agRcvHFeUBQ7BLkVWhttps://1drv.ms/v/s!AglSmr1cGU9agRcvHFeUBQ7BLkVW

SUPPLEMENTARY VIDEO 2: The curtain sign https://1drv.ms/v/s!AglSmr1cGU9agRiGIcTXyRE7A7Lt

SUPPLEMENTARY VIDEO 3: The ski jump sign https://1drv.ms/v/s!AglSmr1cGU9agRlmPhD2KTf8780n

SUPPLEMENTARY VIDEO 4: The lung pulse https://1drv.ms/v/s!AglSmr1cGU9agRomosFYJAwxvrhw

SUPPLEMENTARY VIDEO 5: The substernal access https://1drv.ms/v/s!AglSmr1cGU9agRtjcWcRscUZPJkG
