# Erratum: The Rat Thoracic Ultrasound protocol: scanning technique and normal findings

**DOI:** 10.3389/fvets.2024.1408432

**Published:** 2024-04-11

**Authors:** Frontiers Production Office

**Affiliations:** Frontiers Media SA, Lausanne, Switzerland

**Keywords:** pleura, ultrasonography, rat, RATTUS, lung, respiratory disease, thorax

Due to a production error, the link for Supplementary Video 1 was incorrectly formatted in the article. It has been updated to “https://1drv.ms/v/s!AglSmr1cGU9agRcvHFeUBQ7BLkVW.”

The publisher apologizes for this mistake. The original article has been updated.

