# Supplementary material for: The Rat Thoracic Ultrasound protocol: scanning technique and normal findings
Source: Front Vet Sci. 2024 Feb 19;11:1286614. doi: 10.3389/fvets.2024.1286614 (PMC10909930; doi:10.3389/fvets.2024.1286614)
Supplement: Supplementary file 1 [file Data_Sheet_1.PDF]

For better understanding, detailed description of these ultrasonographic findings is presented in supplementary materials.

The bat sign (sometimes called as the alligator or gator sign; 3) is an abstract sign established to help the examinator with lung surface orientation. The image obtained is called a "bat sign" as the rib heads and pleural line resemble the wings and a body of a bat. The pleura is a bright hyperechoic cover of lung tissue. In normal conditions when the probe is approximated into the chest wall, ribs are visible as rounded structures with a distal acoustic shadow. A rib surface is smooth and hyperechoic, and any disruption in the cortex is a sign of rib fracture or bone lysis (1).

A-lines are hyperechoic horizontal lines that are equally distant from each other (6). These lines are created by reverberation artifacts when ultrasound waves are reflected between two surfaces: a soft tissue (or fluid) and a gas. As A-lines are created in the soft tissue-air interface, they are visible when the aerated lung is above the pleural line or when air is between two pleural lines (pneumothorax; 3).

The lung sliding is a sonographic representation of pleural movement. The visceral and parietal pleura are normally close together, creating a pleural space that is not sonographically visible under normal conditions. The pleural space contains a small amount of fluid which allows the pleura to slide during breathing (7). This sliding is sonographically visible as slight shimmering across the pleural line. As the patient breathes, the diaphragm contracts while the chest wall and visceral pleura move outwards, which causes the lung to expand and the visceral pleura to slide along the chest wall (parietal pleura). The subsequent movement of the visceral and parietal pleura in different ways produces a typical shimmering called "lung sliding". Colour and power Doppler imaging can be used to confirm lung sliding (8).

The normal curtain sign is produced by aerated lung overlapping soft tissue structures in the thorax. The aerated lung plays the role of the curtain. When the lung is filled with air, it expands and retracts over mediastinal or abdominal organs, obscuring the sonographic view into them. Hidden organs are again visible when the lung contracts during expiration. In a healthy patient, two main curtain signs have been identified – abdominal curtain sign and heart curtain sign (3).

The ski jump sign describes the normal appearance of the ventral border of the chest wall. When the probe is parallel to the ribs, the lungs curve away from the sonogram (creating a ski jump-like sonographic image). The ski jump sign is visible when the chest wall passes ventrally to the sternal muscles or the heart, depending on which part of the chest is evaluated (3).

The lung pulse represents pleural oscillation caused by the heartbeat transmitted to the lung surface. It can be observed independently of respiration and indicates that the parietal and visceral pleura are in close contact. The easiest way to identify the lung pulse is in the lung lobes close to the heart (3). Lung pulse in M-mode creates lines T. T-lines are vertical lines arise from

the pleural line to the bottom or seen from downstairs strictly stops at the plural line, they are narrow equivalent of the lung pulse (9, 10). The presence of a lung pulse and T artifacts in M-mode exclude pneumothorax (3).

Π-lines (like Greek letter pi) are visible in some usually skinny human patients and were described by Lichtenstein in examination performed by convex probe. The A-lines can be numerous, associated with sub-A-lines. A-lines are clearly identified between all these sub-A lines (11). In the case of the examination using a linear probe, this artifact is called in human medicine especially in paediatric publications as the bamboo sign, and it usually have no significance (12, 13).

B-lines are vertical hyperechoic comet tail artifacts arising from the pleural line to the bottom of the sonogram, and moving with lung sliding (9, 14, 15). Micro-convex probes are mostly used in veterinary medicine to perform lung ultrasonography. They are used to confirm the presence of B-lines as the definition of the B-lines was made by Daniel Lichtenstein for convex probe (11). To proof that the B-lines are "true" B-lines it is necessary to change the depth and confirm that B-lines did not terminate before reaching the far field of the ultrasound image (16, 17, 18).

It is important to note that the view below the pleural line does not show anatomical lung tissue, but a reverberation artifact of subcutaneous tissue and muscles displayed above the pleura (19). Some structures of the chest wall (e.g. fascia, blood vessel walls) may be mirrored below the pleural line and should not be confused with lung pathology (3).
